# Enhanced Migratory Ability of Neutrophils Toward Epidermis Contributes to the Development of Psoriasis *via* Crosstalk With Keratinocytes by Releasing IL-17A

**DOI:** 10.3389/fimmu.2022.817040

**Published:** 2022-03-23

**Authors:** Xiu-ting Liu, Zhen-rui Shi, Si-yao Lu, Dan Hong, Xiao-nan Qiu, Guo-zhen Tan, Hui Xiong, Qing Guo, Liangchun Wang

**Affiliations:** Department of Dermatology, Sun Yat-sen Memorial Hospital, Sun Yat-sen University, Guangzhou, China

**Keywords:** neutrophils, migration, psoriasis, keratinocytes, interleukin-17A

## Abstract

Microabscess of neutrophils in epidermis is one of the histological hallmarks of psoriasis. The axis of neutrophil–keratinocyte has been thought to play a critical role in the pathogenesis of psoriasis. However, the features and mechanism of interaction between the two cell types remain largely unknown. Herein, we found that blood neutrophils were increased in psoriasis patients, positively correlated with disease severity and highly expressed CD66b, but not CD11b and CD62L compared to healthy controls. Keratinocytes expressed high levels of psoriasis-related inflammatory mediators by direct and indirect interaction with neutrophils isolated from psoriasis patients and healthy controls. The capacity of neutrophils in provoking keratinocytes inflammatory response was comparable between the two groups and is dependent on IL-17A produced by itself. Neutrophils isolated from psoriasis patients displayed more transcriptome changes related to integrin and increased migration capacity toward keratinocytes with high CD11b expression on cell surface. Of interest, neutrophils were more susceptible to keratinocyte stimulation than to fibroblasts and human umbilical vein endothelial cells (HUVECs) in terms of CD11b expression and the production of ROS and NETs. In conclusion, neutrophils from psoriasis patients gain a strong capacity of IL-17A production and integrins expression that possibly facilitates their abilities to promote production of psoriasis-related inflammatory mediators and migration, a phenomenon likely induced by their interaction with keratinocytes but not with fibroblasts. These findings provide a proof-of-concept that development of new drugs targeting migration of neutrophils could be a more specific and safe solution to treat psoriasis.

## Introduction

Psoriasis is an immune-related skin disease histologically featured by neutrophils accumulation in epidermis known as Munro’s or Kogoj’s microabscess. Neutrophils in psoriatic lesions were assumed undergoing respiratory burst, degranulation, and formation of neutrophil extracellular traps (NETs) and producing a plethora of cytokines (interleukin (IL)-1α, IL-1β, IL-6 and others) that facilitated keratinocyte hyperproliferation and activation. In return, keratinocytes secreted chemokines such as CXCL1, CXCL8 (IL-8), and granulocyte colony-stimulating factor (G-CSF) to attract and further activate neutrophils and eventually resulted in a positive feedback loop to contribute to psoriasis development (1–3). Depletion of neutrophils with antibodies suppressed skin inflammation in murine models of psoriasis (4, 5). Drug-induced agranulocytosis resulted in psoriasis remission (6). Together, these findings strongly suggest neutrophils play an important role in the pathogenesis of psoriasis.

Data from animal and clinical studies indicate that IL-17A, the critical effector cytokine in IL-23/IL-17 immunologic pathway, is the principal driver of psoriatic lesion changes (7). Previously, it was hypothesized that the primary source of IL-17A in psoriasis was T helper 17 (Th17) cells. However, emerging bodies of evidence suggested that innate immune cells, especially neutrophils, are potential cellular sources of IL-17A (8, 9). Treatment of IL-17A inhibitors leads to rapid and extensive clearance of neutrophils, which occurred parallel to the improvement of epidermal changes and clinical signs of psoriasis (10). Therefore, a neutrophil–keratinocyte axis in psoriasis may involve neutrophil-derived IL-17A and is an early target of IL-17A-directed therapies.

The findings revealing the importance of neutrophil raise a more interesting question of whether and how psoriatic neutrophils differ from healthy control neutrophils. Previous research found a consistent pattern of changes in a set of modules associated with neutrophil activation and inflammation by analyzing the blood transcriptional fingerprints from two independent public psoriasis datasets. However, the perturbations in blood transcript abundance were relatively subtle compared to the changes in other autoimmune and auto-inflammatory diseases (11). Thus, neutrophils in psoriatic lesions must be addressed together with those in blood in terms of their contribution to psoriasis development. Therefore, we characterized blood neutrophils and investigated the features and mechanism of the interactions between neutrophils and keratinocytes by adapting an *in vitro* assay in the present study.

## Materials and Methods

### Clinical Samples

The study was approved by the research ethics board of the Sun Yat-sen Memorial Hospital. Peripheral blood samples were obtained from each patient with psoriasis vulgaris at the Sun Yat-sen Memorial Hospital, and informed consent of donors was obtained. Normal control blood samples were collected from age- and sex-matched healthy individuals. The moderate-to-severe disease was defined by the Psoriasis Area Severity Index (PASI) as larger than the score of 10. The patients with active disease enrolled in our study were not treated with any medicine for at least 3 weeks.

### Isolation of Neutrophils From Blood Samples

For RNA and protein extraction, neutrophils were isolated from EDTA-anticoagulated blood using Polymorphprep (Alere technologies, Norway; #AS1114683) by density-gradient centrifugation as previously reported (12). For migration assay, neutrophils were isolated by a magnetic-activated cell sorting method with anti-CD15 microbeads (Miltenyi Biotec, Germany; #130-046-601), as previously reported (13). The purity and viability of neutrophils were more than 95% confirmed by flow cytometer with CD15^+^ singlets.

### Flow Cytometry

Approximately 100 µl EDTA-anticoagulated fresh whole blood sample was incubated with proper amount of the following surface antibodies for 30 min on ice in the dark. Surface antibodies included Brilliant Violet 510-anti-CD45 (BD Biosciences, San Jose, CA, #563204), PE-Cy7–anti-CD14 (Invitrogen, Carlsbad, CA; #2085911), APC-Cy7–anti-CD11b (BD Biosciences, #557754), FITC–anti-CD66b (BD Biosciences, #555724), APC-anti-CD62L (BD Biosciences, #559772); APC-Anti-CD15 (Biolegend, San Diego, CA; #301908). After cell surface staining, cells were added RBC lysis buffer and incubated for 10 min, and then washed twice with FACS buffer (1× PBS with 5% FBS, 0.1% NaN3). For viability analysis of neutrophils, cells were stained with Propidium Iodide (Invitrogen, Carlsbad, CA; #208000000). The suspensions were carried out with flow cytometry and analyzed with FlowJo software (Tree Star, Ashland, OR).

### Coculture System

HaCaT or normal human epidermal keratinocytes (NHEK) (75–80% confluency) were cultured with neutrophils (5 × 10^5^ cells/ml, 2 ml) in direct or indirect contact way using neutrophil-impermeable 0.4 µm pore transwell inserts system (Corning, Corning, NY) for 24 h (HaCaT) or 8 h (NHEK) as previously described (13). HaCaT were cultured with neutrophils at different time points (4, 8, 12, and 24 h) or different densities (1.25, 2.5, 5, 7.5 × 10^5^ cells/ml, 2 ml) for 8 h in direct contact way. For immunofluorescence experiments, neutrophils (5 × 10^5^ cells/ml, 1 ml) were cultured with HaCaT, fibroblasts, and HUVECs for 4 h in a direct contact way. To separately analyze the transcripts of IL-17A from HaCaT cells and neutrophils from the coculture system, floating neutrophils were first collected and then the adherent HaCaT cells were washed three times with 5 ml pre-warmed PBS followed by standard detachment protocol with trypsin.

For IL-17A blockade, Secukinumab (12.5 µg/ml) was added to neutrophils suspension for 1 h, and the suspension was transferred to HaCaT and cocultured for 8 h or 24 h as indicated.

### Neutrophil Migration Assay

HaCaT cells were seeded in the bottom chamber of the transwell migration assay with 3 µm pores, uncoated polyester membrane (Corning, Corning, NY) according to previous literature (14). For some experiments, recombinant CXCL8 protein were added into the medium of bottom chamber at a final concentration of 200 ng/ml (15). The neutrophil suspension (100 µl, 2 × 10^6^ cells/ml) was added to the upper compartment and incubated for 45 or 90 min at 37°C and 5% CO_2_. Both the medium in the upper and bottom chambers were collected and were enumerated using flow cytometry on CD15^+^ singlets within a limited time.

### RNA-Sequencing

Total RNA was extracted from neutrophils cultured for 8 h with or without HaCaT in the indirect transwell system. The quality and purity of total RNA was confirmed using a Nano Drop ND-1000 spectrophotometer (Nano Drop, DE, USA) and a Agilent 2100 Bioanalyzer (Agilent, Santa Clara, CA). Paired-end (PE) libraries were prepared following the illumina paired-end library preparation protocol (Illumina, San Diego, CA), and then sequenced on an Illumina NovaSeq sequencing platform to generate 2 × 150 paired end reads. For RNA-seq reads, quality control was performed by the Trimmomatic program (16). Differential expressed analysis was performed by a featureCounts tool and edgeR software (17). Differentially expressed genes were defined as genes with a threshold value of FDR values ≤0.05 and absolute fold change (FC) ≥2 or ≤0.5 respectively. Gene ontology enrichment analysis was performed on the significantly upregulated genes using a DAVID webtool (https://david.ncifcrf.gov/home.jsp). The GO terms with P-values <0.05 were defined as statistically significant.

### Statistical Analysis

Part of data are presented as median ± 95% CI, and were analyzed with GraphPad Prism Version 8.0 (GraphPad Software, San Diego, CA) with Mann–Whitney test as indicated in the legend. Otherwise, data are presented as mean ± SEM and determined by the Student’s t-test when two conditions were compared, and one-way analysis of variance (ANOVA) followed by a Tukey’s multiple comparisons test when more than two conditions were compared. The correlation analyses were performed by a Spearman’s rank correlation test. Values of P <0.05 were considered statistically significant.

More detailed experimental procedures are described in the **Supplementary Methods**.

## Results

### Blood Neutrophils are Increased and Pre-Activated in Psoriasis Patients

**Figure 1:**
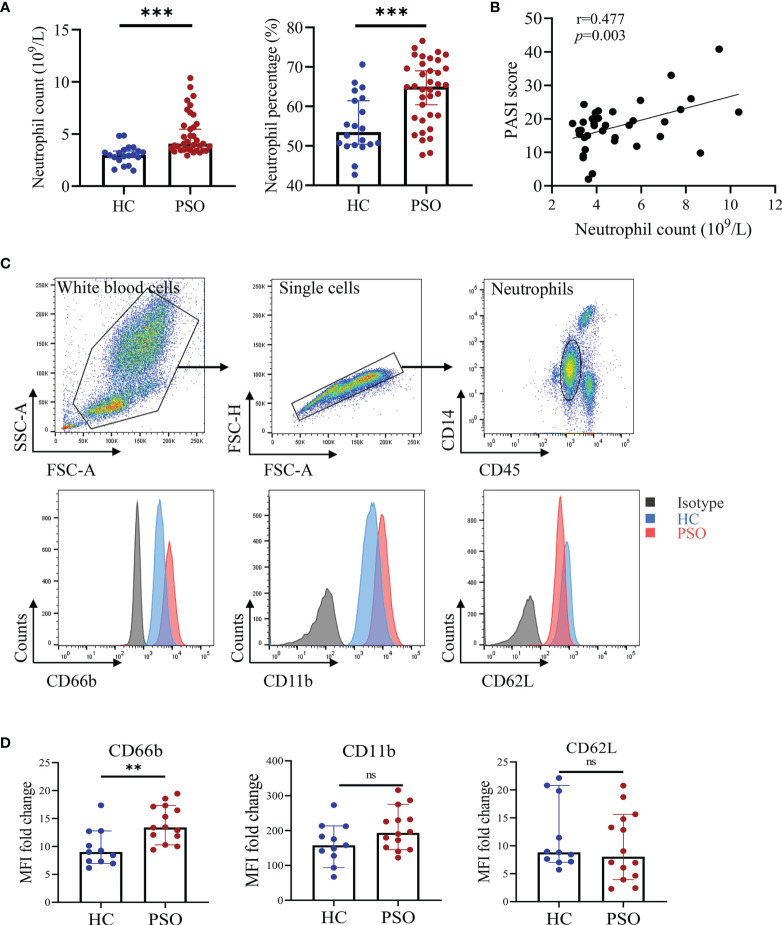
Blood neutrophils are increased and pre-activated in psoriasis patients. **(A)** Absolute numbers and percentages of neutrophils (in white blood cells) in peripheral blood of healthy controls (HC, n = 20) and psoriasis patients (PSO, n = 36). **(B)** The correlation of the absolute numbers of blood neutrophils and PASI score in psoriasis patients (n = 36). **(C)** Representative flow plots showing gating strategy of blood neutrophils and CD66b, CD11b, and CD62L expressions in stained HC (blue) and PSO (red) samples versus isotype control (gray). **(D)** Fold changes of MFI (versus isotype) of CD66b, CD11b, and CD62L on neutrophils of HC (n = 11) and PSO (n = 14). Data are presented as median ± 95% CI. **p < 0.01; ***p < 0.001, by Mann–Whitney test. The correlation analysis was performed by Spearman correlation coefficient. CI, confidence interval; HC, healthy controls; MFI, median fluorescence intensity; ns, not significant; PASI, psoriasis area and severity index; PSO, psoriasis.

We first compared the absolute numbers and percentages of neutrophils in the peripheral blood of psoriasis patients and healthy donors. In line with previous studies (18), the numbers and percentages of blood neutrophils were greatly increased in psoriasis patients compared to healthy subjects (**Figure 1A**). The absolute numbers of neutrophils were positively correlated with disease activities measured by PASI score (**Figure 1B**). Integrin-associated protein CD66b, Mac-1 (CD11b), L-selectin (CD62L) are expressed on neutrophils and govern cell activation and tissue homing (19). We thus assessed CD66b, CD11b, and CD62L expression and found CD66b was markedly increased in neutrophils from the psoriasis patients in comparison to those from the healthy controls (p = 0.0034). CD11b was slightly higher but did not reach a significant difference, while CD62L was comparable between the two groups (**Figures 1C, D**). Together, these results demonstrate that blood neutrophils are increased and present active phenotypes in psoriasis patients.

### Neutrophils Activate Keratinocytes to Produce Psoriasis-Related Inflammatory Mediators

**Figure 2:**
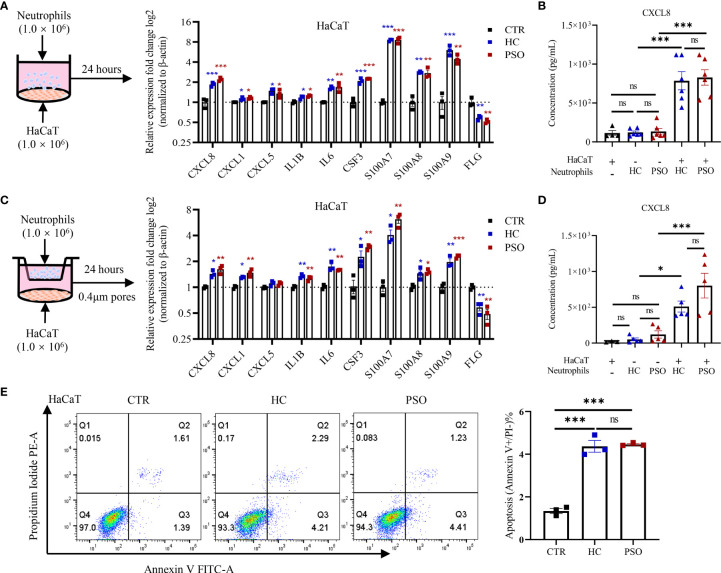
Neutrophils promote inflammatory mediators expression by keratinocytes and cell apoptosis. **(A)** A scheme of direct coculture system (left panel). The mRNA expression of psoriasis-related inflammatory mediators in HaCaT 24 h after direct coculture with neutrophil (right panel). **(B)** Protein levels of CXCL8 in the supernatants determined by ELISA 8 h after co-culture. **(C)** A scheme of indirect coculture system using transwell inserts (left panel). The mRNA expression of psoriasis related-inflammatory mediators (**C**, right panel) and the protein levels of CXCL8 in the supernatants **(D)**. **(E)** Apoptosis rate of HaCaT cells cultured with neutrophils in indirect contact system for 24 h. CTR (control) refers to HaCaT cells without neutrophils. The p-values depicted as asterisk in **(A, C)** refers to comparisons with control. Data are representative of three or more independent experiments. Data are presented as mean ± SEM. *p < 0.05; **p < 0.01; ***p < 0.001, by one-way ANOVA. ELISA, enzyme-linked immunosorbent assay; ns, not significant.

Neutrophils are recruited into psoriatic lesions, particularly in epidermis, where they form into spongiform pustules known as Kogoj in the stratum spinosum and Munro’s microabscesses in the stratum corneum (20). To illustrate the crosstalk of neutrophils and keratinocytes in psoriasis, we cocultured HaCaT cells, an immortalized human keratinocyte line with neutrophils isolated freshly from healthy controls and psoriasis patients for 24 h (**Figure 2A**). Not surprisingly, the mRNA expression of a variety of inflammatory mediators by HaCaT cells was greatly increased, namely, chemokines (CXCL1, CXCL5, CXCL8), cytokines (IL-1B, IL-6, G-CSF), and antimicrobial peptides (AMPs) (S100A7-9). Exposure to neutrophils also led to suppressed expression of filaggrin, a key marker for terminal differentiation of keratinocytes, which was decreased in the psoriatic epidermis (21). However, no differences were observed on these mediators produced by HaCaT cells cocultured with neutrophils from healthy controls and psoriasis patients. Consistent with mRNA expression, CXCL8 protein was elevated in supernatants from HaCaT cells with neutrophil stimulation (**Figure 2B**). We then asked whether or not the response of keratinocytes to neutrophils required direct cell–cell contact. To answer that question, we adapted a transwell cell culture system (**Figure 2C**) to examine the response of keratinocytes as above did. Interestingly, the response of HaCaT cells did not vary with the cell culture system (**Figures 2C, D**), indicating that soluble mediators were sufficient to promote the response of keratinocytes. Similar to what was observed in the direct coculture assay, HaCaT cells responded to neutrophils isolated from psoriasis and healthy control with no differences. We further examined HaCaT cells response at 4, 8, and 12 h after coculture and found consistent upregulations of psoriasis-related inflammatory mediator but with no differences between the two groups at each time points (**Supplementary Figure 1A**). Also, we cultured HaCaT cells with titrated neutrophils (2.5/5/10/15 × 10^5^ cells/ml) and examined their response at 8 h. Again, inflammatory mediators were upregulated even at the lowest titration but was no different between patients and controls (**Supplementary Figure 1B**). We further validated the interactions using primary human epidermal keratinocytes and found the response pattern was not obviously different between HaCaT and NHEK (**Supplementary Figure 2**). In addition to cytokine production, HaCaT cells underwent early apoptosis when indirectly exposed to neutrophils and showed no difference in the apoptotic rate between the two groups (**Figure 2E**). Together, these results demonstrate that blood neutrophils isolated from psoriasis patients and healthy controls harbor similar capacities in promoting keratinocyte responses.

### Neutrophils Promote Keratinocyte Response Through Release of IL-17A

**Figure 3:**
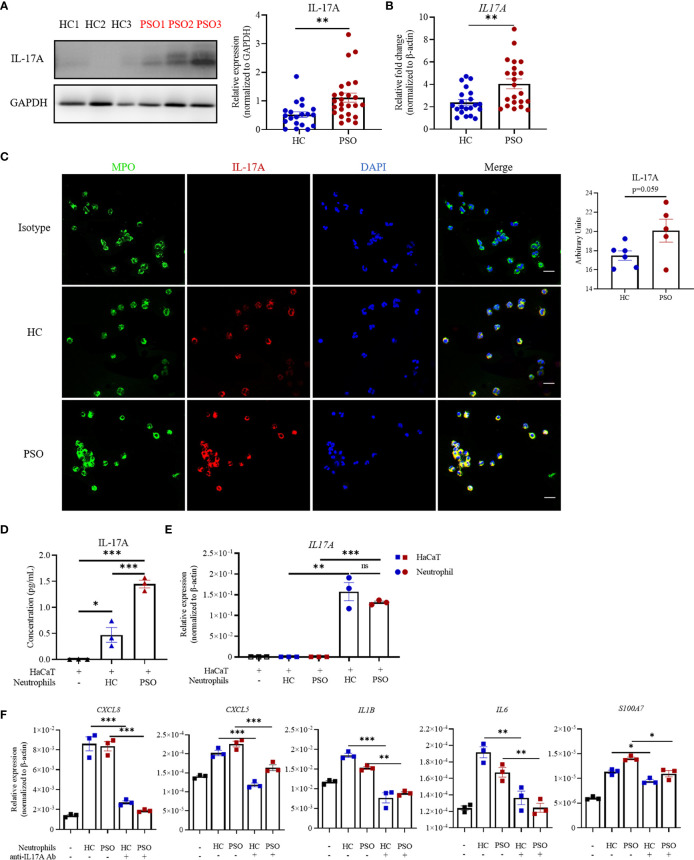
Neutrophils promote the inflammatory response of keratinocytes through release of IL-17A. **(A)** Representative image of IL-17A expression in blood neutrophils examined by western blot (left panel) and quantification of western blot analysis from healthy controls (HC, n = 20) and psoriasis patients (PSO, n = 25) (right panel). **(B)** mRNA expression of IL-17A in blood neutrophils from HC (n = 21) and PSO (n = 22). **(C)** Representative immunofluorescence images of MPO (green),IL-17A (red) and DAPI (blue) expression in blood neutrophils from HC (n = 6) or PSO (n = 5), scale bar = 20 µm. Quantification analysis of IL-17A expression in neutrophils from HC and PSO. **(D)** Protein levels of IL-17A in the supernatant determined by ELISA at 8 h from coculture system of HaCaT alone, HaCaT plus healthy neutrophils or HaCaT plus psoriatic neutrophils. **(E)** mRNA expression of IL-17A in HaCaT cells stimulated by neutrophils (square) and in neutrophils (circle) from psoriasis patients. **(F)** mRNA expression of inflammatory mediators in HaCaT 8 h after directly coculture with neutrophils with and without anti-IL-17A Abs (12.5 μg/ml). Data are representative of three or more independent experiments. Data are presented mean ± SEM and analyzed by the two-tailed Student’s *t*-test or one-way ANOVA. *p < 0.05; **p < 0.01; ***p < 0.001; Abs, antibodies; ns, not significant.

The above-mentioned findings suggested that soluble components were sufficient to promote keratinocyte response. Recent studies highlighted neutrophils as one of the major sources of IL-17A (10). We therefore hypothesized that IL-17A might serve as an important messenger in mediating the crosstalk between keratinocytes and neutrophils in the context of psoriasis. To test this hypothesis, we first measured IL-17A expression in blood neutrophils by Western blot and found that the protein expression of IL-17A was more enriched in neutrophils from psoriasis patients than those from healthy controls (**Figure 3A**). So was the mRNA expression (**Figure 3B**). Also, cell-immunofluorescence of neutrophils, verified the co-localization of IL-17A with myeloperoxidase (MPO), a major enzymatic content of granules specifically expressed in neutrophils (**Figure 3C**) (22). Albeit not statistically significant, there was a trend towards higher intensity of IL-17A in psoriatic neutrophils than healthy neutrophils. Consistently, IL-17A in supernatants was higher when HaCaT cells were cultured with neutrophils from psoriasis patients than those from controls (**Figure 3D**). To evaluate the sources of IL-17A in supernatants, we examined mRNA expression of IL-17A in keratinocytes and neutrophils in direct coculture system. The transcripts of *IL17A* can be only detected in neutrophils but not keratinocytes *in vitro* assay (**Figure 3E**). To conclusively demonstrate the contribution of IL-17A to keratinocyte response stimulated by neutrophils, we performed a coculture assay in the presence of antibodies against IL-17A. Blockade of IL-17A profoundly curtailed the transcripts of proinflammatory markers, namely, CXCL5, CXCL8, IL-1β, IL-6, and S100A7 in HaCaT cells regardless of the sources of neutrophils (**Figure 3F**). Collectively, these findings demonstrate that neutrophils promote keratinocyte response by release of IL-17A.

### Neutrophils Isolated From Psoriasis Patients Harbor Strong Migratory Capacity

**Figure 4:**
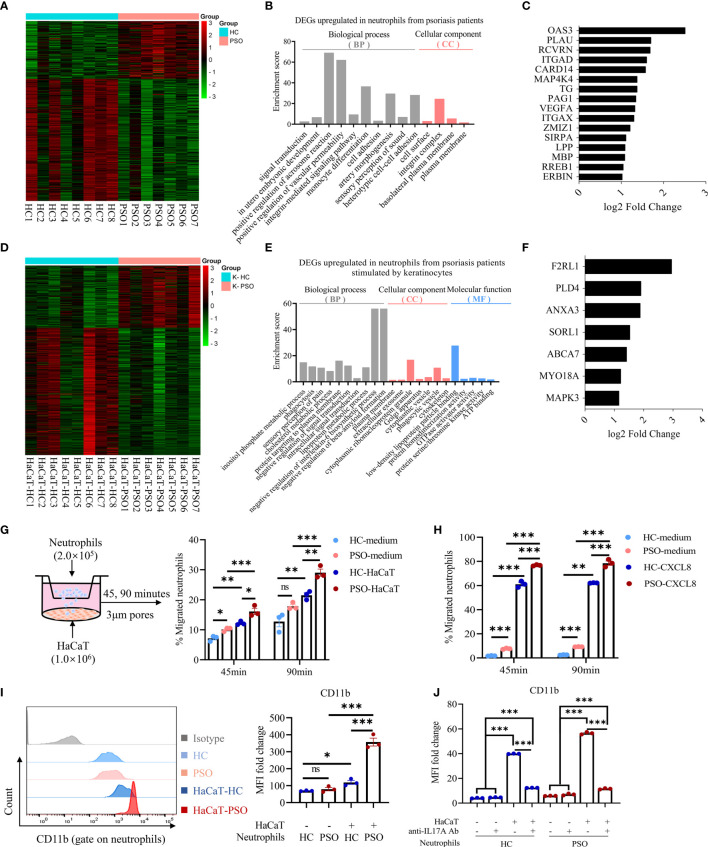
Neutrophils gain migration phenotype and capacity after coculture with keratinocytes. **(A)** Heatmap showing DEGs of RNA-Seq results in PSO (n = 7) versus HC (n = 8) neutrophils cultured alone for 8 h. **(B)** GO category and **(C)** migration-related DEGs upregulated in PSO neutrophils. **(D)** Heatmap showing DEGs in PSO versus HC neutrophils after indirect coculture with HaCaT for 8 h. **(E)** GO category and **(F)** phagocytosis-related DEGs upregulated in PSO neutrophils. **(G)** A scheme of transwell assay. Peripheral blood neutrophils (2 × 10^5^ cells) in the upper chamber were allowed to migrate through 3 μm pores of transwell membrane towards the lower chamber with complete medium or pre-seeded with HaCaT, and the rates of neutrophils migration at indicated time points were analyzed by flow cytometry. HC-medium, HC neutrophils with medium, PSO-medium, PSO neutrophils with medium, HC-HaCaT, HC neutrophils with HaCaT, PSO neutrophils with HaCaT. **(H)** Transwell assay comparing the migration of HC or PSO neutrophils towards complete medium with or without CXCL8 protein (200 ng/ml). **(I)** Representative images of flow cytometry and quantification of MFI fold changes of CD11b expression on neutrophils cultured with or without HaCaT in an indirect manner for 24 h. **(J)** Quantification of MFI fold changes of CD11b expression on neutrophils cultured as described in **(I)** with or withoutanti-IL-17A Abs (12.5 μg/ml). Data are presented as mean ± SEM, one-way ANOVA. *p < 0.05; **p < 0.01; ***p < 0.001. DEGs, differently expressed genes; GO, Gene Ontology; MFI, median fluorescence intensity; ns, not significant; RNA-Seq, RNA-sequencing.

To extensively explore the underlying differences of neutrophils isolated from healthy controls and psoriasis patients, we performed RNA sequencing (RNA-seq) and analyzed the mRNA transcriptome of blood neutrophils from 8 healthy controls and 7 psoriasis patients indirectly cultured with and without HaCaT cells for 8 h. With no stimulation of keratinocytes, neutrophils presented 327 differentially expressed genes (DEGs) that could discriminate the differences of neutrophils between psoriasis patients and healthy controls (FC ≥2 or ≤0.5, respectively; FDR ≤0.05). Of them, 105 were expressed in higher levels and 222 were in lower levels in psoriasis patients (**Figure 4A**). Gene ontology (GO) analysis of the significantly upregulated mRNA demonstrated that these genes could be assigned into several migration-related biological categories such as “positive regulation of vascular permeability”, “integrin-mediated signaling pathway”, “cell adhesion”, and “integrin complex” (**Figure 4B**). The migration-related biological categories of upregulated DEGs included integrins (ITGAD (CD11d), ITGAX (CD11c)), ligand for integrin (VEGFA) and others (**Figure 4C**). With stimulation of keratinocytes, neutrophils presented 491 DEGs. Of them, 161 genes were upregulated and 330 downregulated in neutrophils from psoriasis patients compared to those from healthy controls (FC ≧2 or ≦0.5, respectively; FDR ≦0.05) (**Figure 4D**). GO enrichment analysis revealed that the upregulated genes in neutrophils from psoriasis patients were enriched in biological categories including “phagocytosis” and several metabolic processes (**Figure 4E**). The upregulated DEGs related to phagocytosis function included F2RL1, ANXA3, SORL1, ABCA7 and others (**Figure 4F**). Of interest, we identified 3 overlapping DEGs (HEATR6, HLA-DRB5, and MRPL54) between psoriasis neutrophils with and without stimulation of keratinocytes in relative to healthy neutrophils (**Supplementary Figure 3**). Overall, these findings indicated that blood neutrophils from psoriasis patients might exhibit stronger migratory capacity compared to those from healthy controls. Therefore, we evaluated the migration capacity of neutrophils using a transwell assay (**Figure 4G**). Strikingly, a larger number of neutrophils from psoriasis patients migrated into the low chamber examined at 45 min but reached to a comparable level between patients and controls at 90 min after seeding in the absence of HaCaT cells. More neutrophils migrated into the low chamber in the presence of HaCaT cells and neutrophils from psoriasis patients presented greater capacity compared to those from healthy controls (**Figure 4G**). Consistently, compared to healthy neutrophils, more psoriatic neutrophils were attracted to recombinant CXCL8 protein, one of the most potent neutrophil chemoattractants (23), supporting the stronger migration of psoriatic neutrophils (**Figure 4H**). CD11b is an integrin that played a key role in the adherence of neutrophils to endothelial cells and keratinocytes (24). CD11b expression in neutrophils was comparable between psoriasis patients and controls without keratinocytes stimulation, but was greatly increased after coculture with keratinocytes and was more prominent in psoriasis patients (**Figure 4I**). Blockade of IL-17A strikingly inhibited the elevated expression of CD11b on both healthy and psoriatic neutrophils after exposure to HaCaT cells (**Figure 4J**). Collectively, these data demonstrate that neutrophils isolated from psoriasis patients are featured by strong migration capacity.

### Neutrophils Interact With Keratinocytes With Particular Features

**Figure 5:**
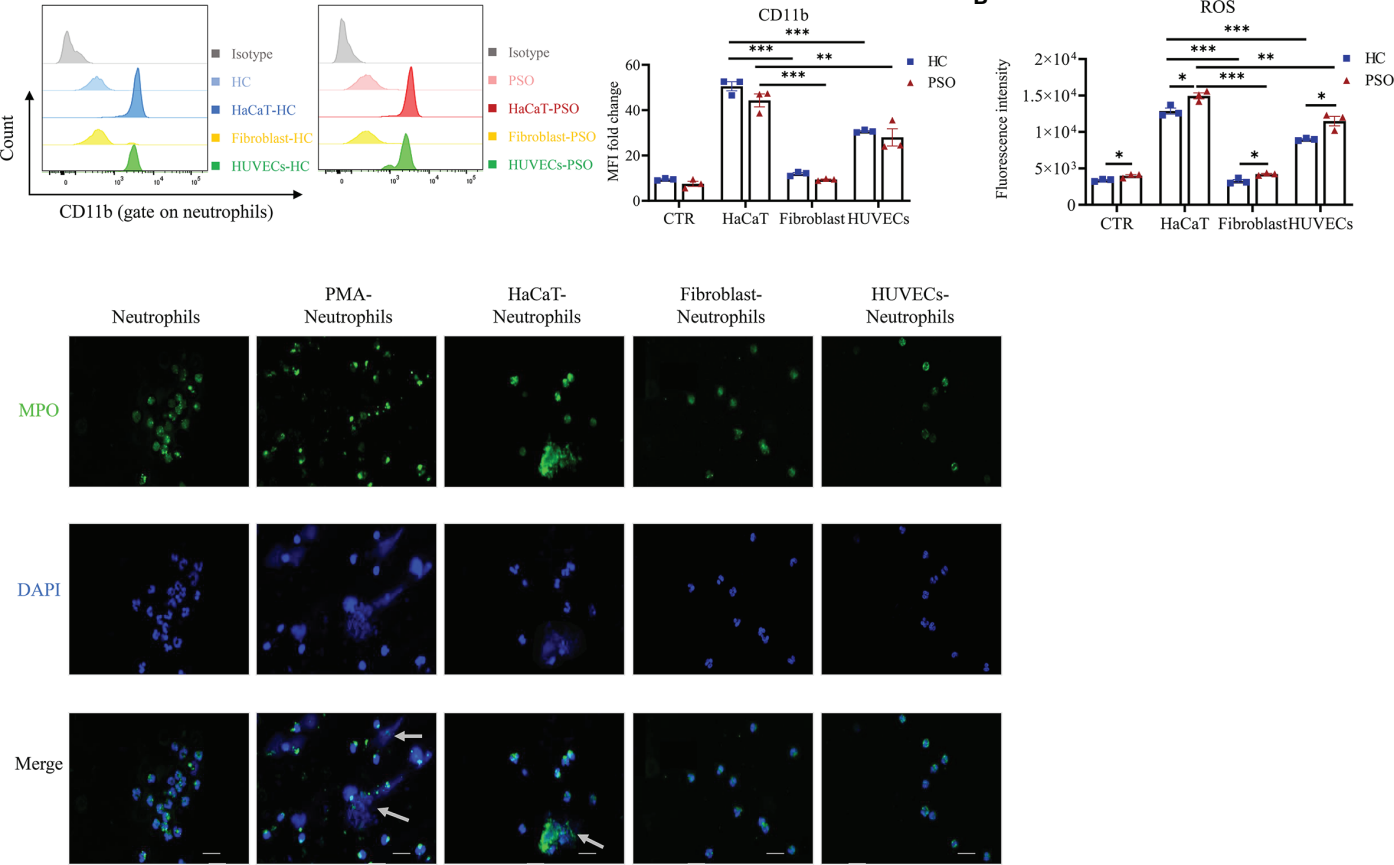
Neutrophils are particularly activated by keratinocytes. **(A, B)** Representative graph and quantification of CD11b expression **(A)** and ROS **(B)** in neutrophils stimulated by HaCaT, fibroblast and HUVEC. **(C)** Representative immunofluorescence staining for MPO (green), DAPI (blue) and merged images of neutrophils stimulated by vehicle, PMA (100 nmol/ml, positive control), HaCaT, fibroblasts and HUVECs, respectively. Scales bar = 20 µm. White arrow indicates NETs. Data are representative of three or more independent experiments. Data are presented as mean ± SEM, analyzed by one-way ANOVA. *p < 0.05; **p < 0.01; ***p < 0.001. HUVEC, human umbilical vein endothelial cells; NETs, neutrophil extracellular traps; ROS, reactive oxygen species.

Our results so far showed that neutrophils isolated from psoriasis patients and healthy controls presented comparable capacity in promoting keratinocytes inflammation, and achieve enhanced capacity of IL-17A production and cell migration in response to keratinocyte stimulation, although that was more prominent in neutrophils from patients. These findings raised an interesting question of whether the interaction pattern of neutrophils with keratinocytes was also applicable to the other cell lines. To answer that question, we cocultured neutrophils with human umbilical vein endothelial cells (HUVECs) and dermal fibroblasts, respectively. We found that neutrophils showed comparable capacity in promoting inflammatory mediators production by HaCaT cells and HUVECs, but less extent by fibroblasts (data not shown). Again, no differences were observed on neutrophils isolated from psoriasis patients and healthy controls in terms of their capacity to stimulate inflammatory mediators production by each cell lines. Neutrophils co-cultured with HaCaT cells expressed higher level of CD11b than those with fibroblasts and HUVECs (**Figure 5A**). Recent reports suggested that the reactive oxidative stress (ROS) and NETs produced by neutrophils were related to the initial and maintenance phases of psoriasis (25–27). In support of the findings, we found ROS production was more rigorous in neutrophils stimulated by HaCaT cells than those by fibroblasts and HUVECs at 8 h after co-culture (**Figure 5B**). Psoriatic neutrophils exhibited moderately higher levels of ROS than healthy neutrophils regardless of the cocultured cells. The formation of NETs was defined as the colocalization of DNA and MPO in laminar extracellular stretches examined by immunofluorescence assay. We next investigate the formation of NETs induced by various types of cells. Neutrophils incubated with PMA (phorbol 12-myristate 13-acetate) was taken as a positive control (28), while unstimulated neutrophils serve as negative control. As shown in **Figure 5C**, NETs formation was observed in neutrophils cocultured with keratinocytes and less likely found with fibroblast and HUVECs. Together, these data showed that neutrophils are more susceptible to keratinocyte stimulation than to endothelial cells and fibroblasts.

## Discussion

In the present study, we showed that (1): The absolute numbers of blood neutrophils was elevated in psoriasis patients than in healthy controls and positively related to psoriasis severity (2). Neutrophils from psoriasis patients expressed higher levels of CD66 and IL-17A, promoted psoriasis-related inflammatory mediators production by keratinocytes in an IL-17A-dependent manner but with no differences from healthy controls (3). Neutrophils from psoriasis patients harbored a panel of abundance transcriptomic integrins and facilitated its migration toward keratinocytes (4). Neutrophils showed priority in response to keratinocyte stimulation determined by strong CD11b expression, ROS production, and NETs formation.

It is well known that keratinocytes attract neutrophils by releasing CXCL-1 and CXCL-8 expression in the context of psoriasis (29). We observed that the major chemotactic molecules expressed by keratinocytes were comparable regardless of their direct and indirect interaction with neutrophils isolated from psoriasis patients and healthy controls. However, neutrophils from psoriasis patients did migrate more vigorously toward keratinocytes than those from controls in the transwell assay. These findings implicated that the enhanced migration of neutrophils was possibly due to its intrinsic capacity rather than merely to chemokine attraction in psoriasis. In agreement with the hypothesis, we observed that neutrophils freshly isolated from psoriasis patients presented more gene enrichment in integrin-related biological categories. Indeed, the expression of CD11b, one of the key leukocyte integrins required for adhesion and migration (30), was upregulated significantly on neutrophils isolated from psoriasis in response to keratinocyte stimulation. Thus, our data suggested that blood neutrophils from psoriasis are predisposed to be activated, consequently express higher integrins levels and enhance migration to keratinocytes. Of interest, a recent study showed that neutrophils from psoriasis patients significantly increased the permeability of cutaneous vascular endothelial cells by the release of MMP-9 (31). Together, neutrophils are prone to migrate into inflamed skin lesions *via* multiple mechanisms in psoriasis.

It was long thought that the primary source of IL-17A in psoriasis was Th17 cells. Increased numbers of Th17 cells are found in the blood and affected skin of patients with psoriasis (32, 33). Recently, however, there has been a paradigm shift in the understanding of cellular sources of IL-17A in psoriasis. Increasingly, data indicate that additional important cellular sources of IL-17A are neutrophils, mast cells, αβ Tcells, γδ T cells, and innate lymphoid cells (7). Dual-color immunofluorescence showed that IL-17A^+^ neutrophils considerably outnumbered IL-17A^+^ T cells in the lesional skin (34), suggesting neutrophils as an abundant source of IL-17A in psoriasis. Early clinical response to secukinumab was linked to the disappearance of cutaneous neutrophils, while their reoccurrence preceded the clinical relapse (10). Notably, the decrease of neutrophils in skin and normalization of epidermis occurred prior to the changes of other immune cells, including T cells and CD11c-positive dendritic cells, supporting neutrophil–keratinocyte axis as an early target of IL-17A-directed therapies. Consistent with these clinical observations, our *in vitro* data demonstrated that IL-17A from neutrophils was indispensable for their capacity to induce inflammation in keratinocytes. Unexpectedly, neutrophils from both healthy donors and psoriasis patients similarly activate keratinocytes despite higher expression of IL-17A in psoriatic neutrophils. We hypothesize that IL-17A mediates the proinflammatory function of neutrophils in our coculture system on keratinocytes in a dose-independent manner. Previous studies have suggested that IL-17 appears insufficient to mount a robust inflammatory response by itself. Instead, IL-17A interacted with other cytokines such as tumor necrosis factor-α or IL-1β synergistically to exaggerate their influence (35). It is a possibility that IL-17A at low concentration was sufficient to orchestrate a full inflammatory cascade. In accordance with this theory, we showed that the inflammatory phenotype of keratinocytes induced by healthy or psoriatic neutrophils was comparably suppressed by IL-17A blockade. To be noted, other mechanisms beyond IL-17A also contribute to the complex communication between keratinocytes and neutrophils in psoriasis. For instance, cytokine-treated keratinocyte exosomes significantly induced NETosis and the expressions of IL-6, IL-8, and TNF-a in normal neutrophils (36). In future studies, it will be of interest to compare the activity of psoriatic neutrophils stimulated with non-cytokine-treated or cytokine-treated keratinocytes.

Several studies showed that neutrophils in psoriatic lesions were co-localized with IL-17A (10, 34). However, a recent study displayed that highly purified human neutrophils did not express IL-17A or other IL-17 family cytokines *in vitro* (37). We showed here the abundance of IL-17A mRNA and protein expression by blood neutrophils isolated from psoriasis patients with and without keratinocyte stimulation and by neutrophils from healthy controls only under the situation of keratinocyte stimulation. The discrepancy could be due to the experimental settings and the patient cohorts as well. In concordance with previous research (38), our data showed that keratinocytes barely produced IL-17A, suggesting neutrophils are the major sources of IL-17A in *in vitro* assay. Of note, a limitation of our studies was the dependence on techniques that drive the cells into a highly activated state using culture methods. Further studies using *in vivo* models for psoriasiform dermatitis or other IL-17 driven disease are warranted to comprehensively investigate the cellular source of IL-17A.

A previous study suggested that neutrophil-to-lymphocyte ratio (NLR) was related to vascular dysfunction including lower aortic velocity propagation (AVP) and higher carotid intima-media thickness (CIMT) values (39). Blood neutrophils from psoriasis shared similar transcriptome signatures with those from Kawasaki disease, an acute systemic vasculitis mainly affecting coronary arteries. In accordance, the vascular dysfunction was closely related to neutrophil infiltration into the aortic vessel wall and serum IL-17A levels in a murine psoriasis model (40). In our study, coculture of neutrophils with HUVEC triggered HUVEC inflammation and, to less extent, induced neutrophil activation determined by ROS production, CD11b expression, and formation of NETs. Thus, one would assume the pre-activation of neutrophils might partially contribute to cardiovascular disorders in psoriasis patients.

In conclusion, our data suggest that neutrophils from psoriasis patients wear disease features in terms of IL-17A and integrins overexpression upon crosstalk with keratinocytes, which might be responsible for neutrophils-induced inflammatory mediators production and accumulation of neutrophils in the epidermis. These findings provide a proof-of-concept that the discovery of new drugs targeting migration of neutrophils is a more specific and safe solution to treat psoriasis.

## Data Availability Statement

The datasets presented in this study can be found in online repositories. The names of the repository/repositories and accession number(s) can be found below: https://www.ncbi.nlm.nih.gov/, sra/PRJNA780945.

## Ethics Statement

The studies involving human participants were reviewed and approved by the research ethics board of the Sun Yat-sen Memorial Hospital. The patients/participants provided their written informed consent to participate in this study.

## Author Contributions

XL, DH, SL, and XQ performed and analyzed the experiments. ZS, QG, and LW drafted the manuscript, designed the experiments, and reviewed the manuscript. GT contributed conceptually to the project and assisted in manuscript preparation. HX assisted in collecting clinical samples and manuscript preparation. All authors listed have made a substantial, direct, and intellectual contribution to the work and approved it for publication.

## Funding

This study was supported by the National Natural Science Foundation of China [grants 82073431, 81872524], the Guangdong Basic and Applied Basic Research Foundation [grant numbers 2020A1515110320], and the Sun Yat-Sen Clinical Research and Cultivation Project [grant number SYS-C-202005].

## Conflict of Interest

The authors declare that the research was conducted in the absence of any commercial or financial relationships that could be construed as a potential conflict of interest.

## Publisher’s Note

All claims expressed in this article are solely those of the authors and do not necessarily represent those of their affiliated organizations, or those of the publisher, the editors and the reviewers. Any product that may be evaluated in this article, or claim that may be made by its manufacturer, is not guaranteed or endorsed by the publisher.

## Abbreviations

Abs, antibodies; DEGs, differently expressed genes; IL, interleukin; HC, healthy control; HUVEC, human umbilical vein endothelial cells; KCs, keratinocytes; MPO, myeloperoxidase; NETs, neutrophil extracellular traps; PASI, Psoriasis Area Severity Index; RNA-seq, RNA sequencing; ROS, reactive oxidative stress; Th17, T helper 17.
