# Supplementary material for: Enhanced Migratory Ability of Neutrophils Toward Epidermis Contributes to the Development of Psoriasis via Crosstalk With Keratinocytes by Releasing IL-17A
Source: Front Immunol. 2022 Mar 23;13:817040. doi: 10.3389/fimmu.2022.817040 (PMC8983831; doi:10.3389/fimmu.2022.817040)
Supplement: Supplementary file 1 [file DataSheet_1.pdf]

Supplementary Figures

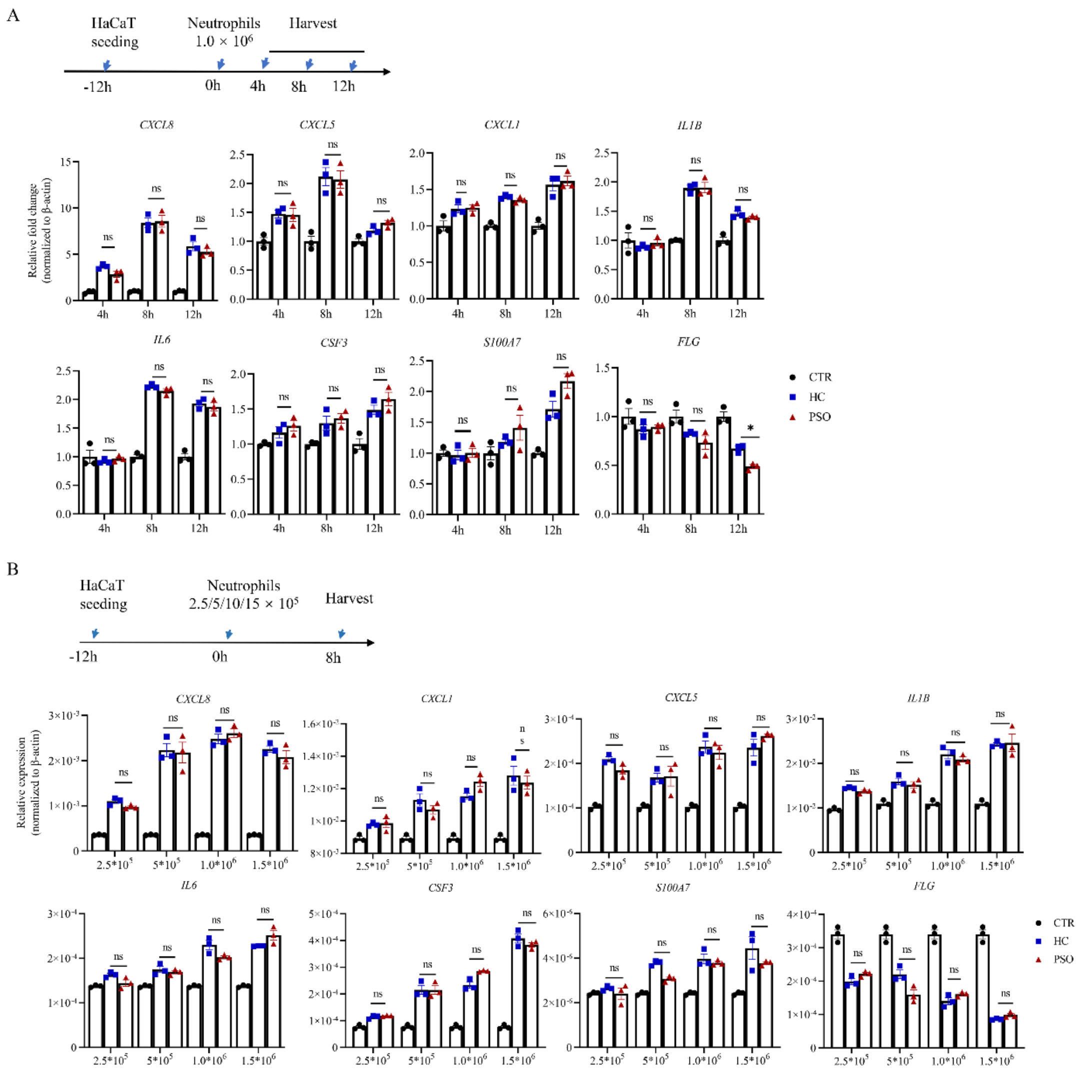

Supplementary figure 1. Psoriatic and healthy control neutrophils induce comparable extent of inflammation mediators in keratinocytes.

(A) The mRNA expression of psoriasis-related inflammatory mediators in HaCaT cultured with HC and PSO neutrophils in direct manner at different time points (4,8,12 hours) according to the experimental scheme shown (upper panel). (B) The mRNA expression of psoriasis-related inflammatory mediators in HaCaT cultured with different densities (2.5, 5, 5, 10 × 10^5 cells) of neutrophils for 8 hours in direct coculture according to the experimental scheme shown. Data are representative of three independent experiments. Data are presented as mean ± SEM and statistical

analysis was performed using one-way ANOVA. *$p< 0.05$; **$p< 0.01$; ***$p< 0.001$, ns, not significant.

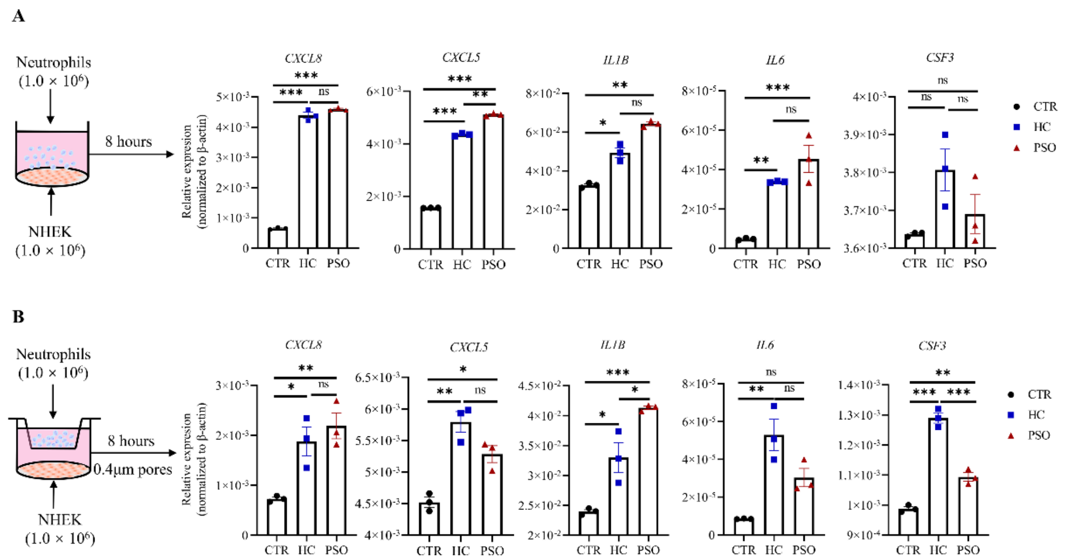

Supplementary figure 2. Neutrophils induce proinflammatory phenotype in primary keratinocytes

(A) A scheme of direct coculture system (left panel). The mRNA expression of psoriasis-related inflammatory mediators in primary keratinocytes cell (Normal human epidermal keratinocytes, NHEK) after 8 hours cultured with neutrophil (right panel). (B) A scheme of indirect coculture system using Transwell inserts (left panel). The mRNA expression of psoriasis related-inflammatory mediators in primary keratinocytes cell after 8 hours indirect cultured with neutrophil (right panel). Data are presented as mean ± SEM and statistical analysis was performed using one-way ANOVA. *$p< 0.05$; **$p< 0.01$; ***$p< 0.001$, ns, not significant.

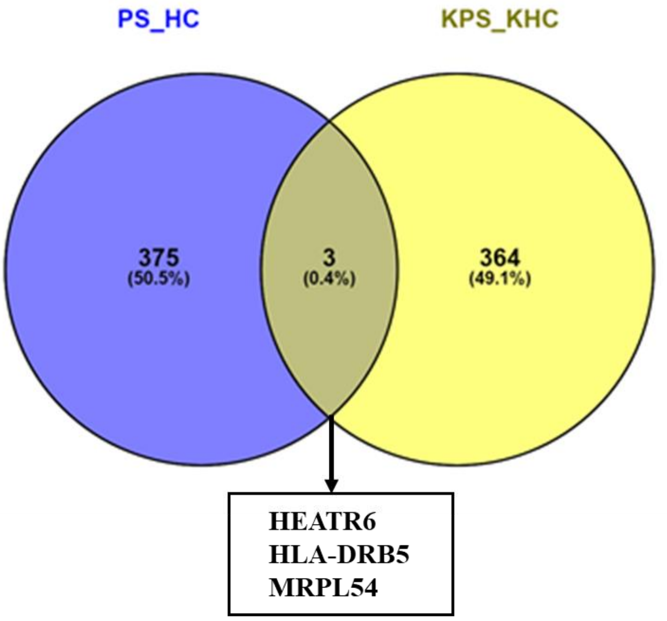

Supplementary figure 3. A Venn diagram of 3 overlapping DEGs in psoriatic neutrophils with and without stimulation. PS_HC, psoriatic neutrophils vs healthy neutrophils； KPS_KHC, psoriatic neutrophils vs healthy neutrophils in contact with HaCaT keratinocytes.

Supplementary Materials and Methods

Cell culture

HaCaT (IBS, China) were cultured in DMEM supplemented with 10% fetal bovine serum (FBS), 10 U/mL of penicillin, and 10 μg/mL of streptomycin.

Normal human epidermal keratinocytes (NHEK) (ATCC, Virginia, USA) were cultured in Dermal Cell Basal Medium (ATCC) supplemented with Keratinocyte Growth Kit (ATCC). Dermal fibroblasts were isolated from normal healthy skin and were cultured in DMEM supplemented with 10% FBS as previously reported (1).

Human umbilical vein endothelial cells (HUVECs) (ATCC) were cultured in Endothelial Cell Medium (ScienceCell, San Diego, California; #1001) consisting of 5% FBS. Blood neutrophils were isolated and suspended in RPMI 1640 medium with 10% FBS. All the cells were cultured in a humidified, 5% CO_2 incubator at 37 °C.

Quantitative real-time PCR

Total RNA was extracted from neutrophils and keratinocytes using EZ-press RNA Purification Kit (EZBioscience, China; #B0004DP) according to the manufactures' instructions. Complementary DNA was synthesized using Color Reverse Transcript Kit (EZBioscience, #A0010CGQ). RT-qPCR was carried out with Roche LightCycler® 480 instrument using SYBR Green Color qPCR Mix (EZBioscience, #A0012-R2).

Western blot assay

Isolated Neutrophils were lysed with a radioimmunoprecipitation assay (RIPA) lysis buffer with protease and phosphatase inhibitors for the total protein extraction as previous studies indicated (2). Antibodies against IL-17A (R&D system, Minneapolis, MN; # AF-317-NA) were used for immunoblot analysis according to the manufactures' protocols. The relative expression of IL-17A was confirmed using the quantity of glyceraldehyde-3-phosphate dehydrogenase (GAPDH). The intensity of the Western blot bands were quantified by Image J software.

Apoptosis assay of keratinocytes

The keratinocytes stimulated by neutrophils for 24 hours in an indirect contact way

were digested and collected. The cells were stained in binding buffer containing annexin V/FITC (eBioscience, #BMS306FI-300) for 20 minutes. Propidium iodide (Invitrogen, #208000000) was added before being analyzed by flow cytometry immediately.

Enzyme-linked immunosorbent assay (ELISA)

The levels of IL-17A and CXCL8 in the supernatants of the coculture system were measured using an ELISA kit (eBioscience, #88-7176-22; ThermoFisher, # 88-8086-22) according to the manufacturer's instructions as previously reported (2).

Detection of Reactive Oxygen Species

The levels of intracellular reactive oxygen species (ROS) produced by neutrophils were measured by fluorescence spectrophotometer using DCFH-DA (Beyontime, China; #S0033S-1) following the manufactures' instructions.

Immunofluorescence

The 6-well plates were pre-seeded with HaCaT, fibroblast, and HUVECs. Blood neutrophils (5×10^5 cells/mL, 2mL) were seeded on poly-L-lysine–coated coverslips and allowed to adhere for 4 hours in the 6-well plates. The protocol for NETs visualization by immunofluorescence was as previously described (3). After stimulation with different cells, neutrophils on the coverslip were stained with anti-MPO antibody (Abcam, Cambridge, United Kingdom; #ab208670, 1:500) and anti-IL-17A antibody (R&D system, #AF-317-NA, 1:200) at 4°C overnight followed by goat anti-rabbit IgG AlexaFluor488 (Abcam, #ab150077) and donkey anti-goat IgG AlexaFluor647 (Absin Bioscience, China; #abs20027) for 1 hours. Nuclear morphology was stained with DAPI (Abcam, #ab188804). Laser scanning confocal microscopy was done using an LSM 710 confocal microscope (Zeiss, Germany). Mean value of fluorescence (arbitrary units, AU) was quantified as integrated density/area using ImageJ program as previously described (4). Six to eight non-overlapping areas were randomly selected from each sample to obtain an average value.

Reference

1. F. Nejaddehbashi, V. Bayati, L. Mashali, M. Hashemitabar, M. Abbaspour, E. Moghimipour and M. Orazizadeh: Isolating human dermal fibroblasts using serial explant culture. *Stem Cell Investig*, 6, 23 (2019) doi:10.21037/sci.2019.08.05
2. Z. R. Shi, G. Z. Tan, C. X. Cao, Y. F. Han, Z. Meng, X. Y. Man, Z. X. Jiang, Y. P. Zhang, N. N. Dang, K. H. Wei, D. F. Bu, F. T. Liu and L. Wang: Decrease of galectin-3 in keratinocytes: A potential diagnostic marker and a critical contributor to the pathogenesis of psoriasis. *J Autoimmun*, 89, 30-40 (2018) doi:10.1016/j.jaut.2017.11.002
3. E. Apostolidou, P. Skendros, K. Kambas, I. Mitroulis, T. Konstantinidis, A. Chrysanthopoulou, K. Nakos, V. Tsironidou, M. Koffa, D. T. Boumpas and K. Ritis: Neutrophil extracellular traps regulate IL-1beta-mediated inflammation in familial Mediterranean fever. *Ann Rheum Dis*, 75(1), 269-77 (2016) doi:10.1136/annrheumdis-2014-205958
4. E. C. Jensen: Quantitative analysis of histological staining and fluorescence using ImageJ. *Anat Rec (Hoboken)*, 296(3), 378-81 (2013) doi:10.1002/ar.22641
